# Autonomic Responses Associated with Olfactory Preferences of Fragrance Consumers: Skin Conductance, Respiration, and Heart Rate

**DOI:** 10.3390/s24175604

**Published:** 2024-08-29

**Authors:** Bangbei Tang, Mingxin Zhu, Yingzhang Wu, Gang Guo, Zhian Hu, Yongfeng Ding

**Affiliations:** 1 Department of Physiology, Army Medical University, Chongqing 400038, China; tangbangbei@126.com; 2 School of Intelligent Manufacturing Engineering, Chongqing University of Arts and Sciences, Chongqing 402160, China; zhumingnix@126.com (M.Z.); dyf_tjl@163.com (Y.D.); 3 School of Mechanical Engineering, Sichuan University of Science & Engineering, Yibin 643000, China; 4 College of Mechanical and Vehicle Engineering, Chongqing University, Chongqing 400044, China; cquwyz@cqu.edu.cn (Y.W.); guogang@cqu.edu.cn (G.G.)

**Keywords:** fragrance products, olfactory preferences, wearable devices, physiological signals, preference evaluation

## Abstract

Assessing the olfactory preferences of consumers is an important aspect of fragrance product development and marketing. With the advancement of wearable device technologies, physiological signals hold great potential for evaluating olfactory preferences. However, there is currently a lack of relevant studies and specific explanatory procedures for preference assessment methods that are based on physiological signals. In response to this gap, a synchronous data acquisition system was established using the ErgoLAB multi-channel physiology instrument and olfactory experience tester. Thirty-three participants were recruited for the olfactory preference experiments, and three types of autonomic response data (skin conductance, respiration, and heart rate) were collected. The results of both individual and overall analyses indicated that olfactory preferences can lead to changes in skin conductance (SC), respiration (RESP), and heart rate (HR). The trends of change in both RESP and HR showed significant differences (with the HR being more easily distinguishable), while the SC did not exhibit significant differences across different olfactory perception preferences. Additionally, gender differences did not result in significant variations. Therefore, HR is more suitable for evaluating olfactory perception preferences, followed by RESP, while SC shows the least effect. Moreover, a logistic regression model with a high accuracy (84.1%) in predicting olfactory perception preferences was developed using the changes in the RESP and HR features. This study has significant implications for advancing the assessment of consumer olfactory preferences.

## 1. Introduction

Fragrance products serve various purposes, such as enhancing mood, creating ambiance, and boosting personal charm. The evaluation of consumer olfactory preferences is an important process for the development and marketing of fragrance products. Olfactory preferences are influenced by various factors, including personal experiences, physiological characteristics, and emotional states [1]. Thus, the establishment of preferences goes beyond cognitive–logical processes [2]. With the deepening of consumer behavior research, traditional surveys and analysis methods are gradually becoming insufficient for a comprehensive understanding of complex consumer psychology.

Evaluation methods for olfactory preferences can be classified into subjective and objective evaluation methods. A subjective evaluation method is to collect subjective feedback on the product during the user’s use process [3]. In subjective evaluation methods, scales are often used to evaluate olfactory perception. Sorokowska et al. [4], Farahani et al. [5], and Klyuchnikova et al. [6] used a Likert scale to evaluate the pleasantness of odors. In an olfactory recognition test, Fjaeldstad et al. [7] and Lesur et al. [8] applied a visual analog scale to assess the intensity and pleasantness of odors. APNEA [9] adopted the Likert scale to investigate the impact of olfactory stimuli on dream perception. Mu et al. [10] developed an 11-point classification scale to evaluate olfactory perception. Zhou et al. [11] used the vividness questionnaire of olfactory image. However, the results of subjective evaluation are unreliable and inaccurate due to the lack of both support from objective data and professional olfactory training among the participants. Objective evaluation methods rely on measuring objective indicators, such as behavioral data [12,13], activity of brain cells and neurons [14,15,16], and hormone levels [17]. Objective evaluation methods can accurately reflect olfactory perception. However, some objective evaluation methods are difficult to apply and require a lot of time and economic expenditure (such as measuring the activity of brain cells and neurons).

As a spontaneous response of the human body, physiological signals are not easily influenced by the subjective consciousness of the subject, thus possessing superb objectivity, accuracy, and validity [18,19,20]. Physiological data is often used to assess physiological and psychological states [21]. Specifically, the human body reflects psychological and physiological conditions through physiological signals when stimulated [22,23]. Therefore, physiological signals can be used as indicators to assess olfactory preferences. In studies related to olfactory stimuli, heart rate data have been used to explore individual preferences for odors [24,25]. Ohira et al. [26] used skin conductance signals to assess the preferences of fragrance consumers. Besides electrocardiogram and skin conductance data, respiratory-related physiological signals are also commonly used to assess the emotional aspect of odor preferences [27,28]. Although some studies have begun to explore the use of physiological signals to assess the olfactory preferences of fragrance consumers, these studies are still in the exploratory stage and lack relevant explanations and applications. In addition, a single physiological signal cannot fully capture the complexity of olfactory preferences [29]. Therefore, it is necessary to combine multiple physiological signals to assess olfactory preferences.

Based on the knowledge gap mentioned above, this study aims to investigate the autonomic responses (skin conductance, respiration, and heart rate) of fragrance consumers when they exhibit olfactory preferences and explore an effective method for evaluating olfactory preferences through a quantitative analysis of autonomic response changes. We collected these physiological signals from the consumers through biosensors and investigated the changes in the physiological signals of the fragrance consumers when they exhibited olfactory preferences. Then, the differences in the physiological signal changes caused by the different olfactory preferences were explored. Furthermore, the influence of gender differences on the results was investigated. Finally, an olfactory preference prediction model was established based on distinguishable physiological signals.

## 2. Materials and Methods

### 2.1. Participants

This study recruited 33 consumers (15 males and 18 females). All participants were between 21 and 32 years of age (M = 26.7 years; SD = 2.6 years). All participants did not have neurological disorders or rhinitis and did not consume strongly scented food before the experiment. Individuals who met all requirements were selected as participants (as is shown in Table 1). The experimental content and procedures of this experiment were approved by the Ethics Committee of Chongqing University of Arts and Sciences (approval no. CQWL202401). Participants’ data were processed following the Declaration of Helsinki. After obtaining the consent of the participant, an informed consent form was signed by the participant to inform them of the experiment content and the tasks to be completed during the experiment.

### 2.2. Equipment and Procedure

In this study, peppermint, jasmine, sweet orange, and lavender essential oils were utilized as odor sources (Refined Aroma, Shanghai, China, so their standardization is ensured, and they are non-toxic and harmless to humans). The concentration of these essential oils is 5%. These scents are extensively utilized in both experiments and daily life [30,31,32]. The generation of odors was achieved through olfactory experience tester [33,34] (Interactive Technology, Chongqing, China). The ErgoLAB signal acquisition module was used to collect and record the physiological signals of participants [35,36] (KingFar International Inc., Beijing, China). The program for the experimental process was written through the ErgoLAB human–computer interaction platform [37] (KingFar International Inc., Beijing, China).

**Figure 1:**
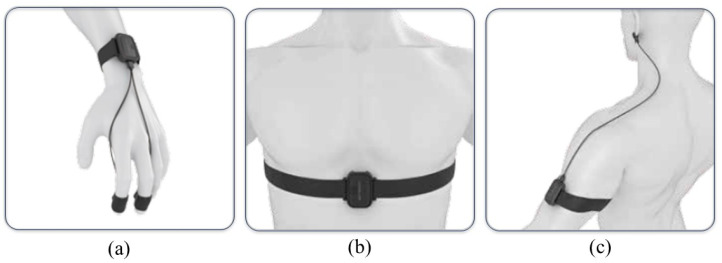
Wearing diagram of physiological signal sensors. (**a**) Wearing diagram of EDA sensor; (**b**) Wearing diagram of RESP sensor; (**c**) Wearing diagram of PPG sensor. User manual of multi-channel physiological instrument was referenced to create (**a**–**c**).

The physiological signal data acquisition equipment is as follows:

(1) ErgoLAB EDA wireless skin conductance sensor (sampling rate: 64 Hz, acquisition range: 0–30 μS). The two electrodes of the EDA sensor are fixed at the fingertips of the index finger and middle finger (as shown in Figure 1a).
(2) ErgoLAB RESP wireless respiratory sensor (sampling rate: 64 Hz; acquisition range: 0–140 rpm). The belt of the RESP sensor is fixed between the chest and abdomen of the subject (as shown in Figure 1b).
(3) ErgoLAB PPG wireless blood volume pulse sensor (sampling rate: 64 Hz; acquisition range: 0–240 bpm). The ear clip electrodes of the PPG sensor are fixed on the earlobe (as shown in Figure 1c).

**Figure 2:**
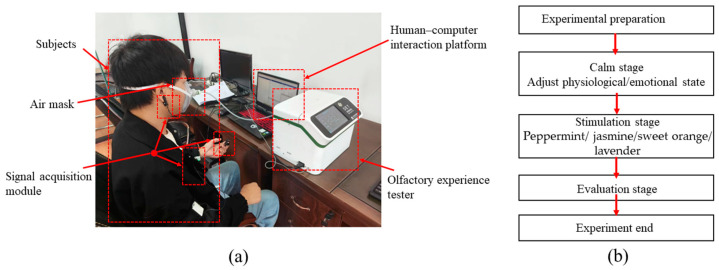
Experimental environment and process. (**a**) Experimental environment. (**b**) Experimental flowchart.

The ErgoLAB human–computer interaction platform was connected to the signal acquisition module and the olfactory experience tester to form a synchronous data acquisition system (as shown in Figure 2a). As the experimental process progresses, the ErgoLAB-controlled olfactory experience tester releases experimental gases. The physiological signals at both the start and end of each experimental phase were marked. The time period between the start and end markers of a stage was used as the time window for dividing the signal. 

**Figure 3:**
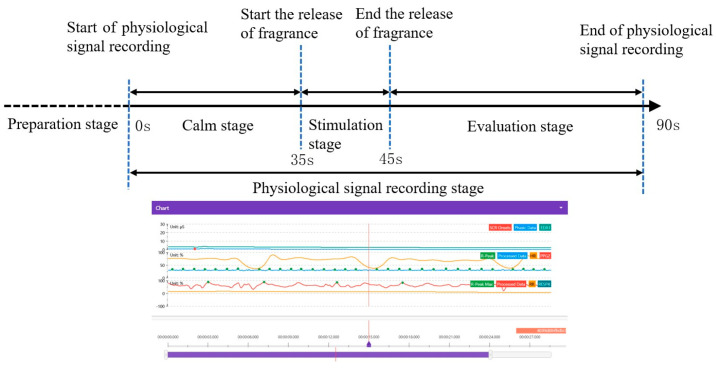
Experimental process and signal segmentation.

Each participant was required to complete four sets of experiments (peppermint, jasmine, sweet orange, and lavender), which were conducted in a random order. These experiments do not interfere with each other. A total of 132 samples were generated from thirty-three participants (as shown in Figure 2b). Each experiment was divided into four stages: the preparation stage, calm stage, stimulation stage, and subjective evaluation stage (as shown in Figure 3). During the preparation stage, participants were fitted with an odor mask and a physiological signal acquisition module, both of which were securely placed to avoid interfering with the participant’s movements. The experiment assistant then helped the participant adjust to a comfortable and sustainable posture. Once the participant was ready, physiological signals were recorded for 95 s. The first 35 s represent the calm stage, during which the participant’s physiological signals are maintained within a normal range. The physiological signals during the calm stage were used as a baseline, and each participant’s physiological signals were only compared to their own baseline, eliminating the impact of individual differences. From the calm stage, participants were asked to remain still and not speak, to ensure that the signal changes during the stimulation stage were caused by the fragrance. From 35 s to 45 s, an odor was released, allowing the participant to experience experimental odors. Finally, from 45 s to 95 s, the subjective evaluation of the participant was collected. This evaluation had two indicators: “like” and “disgust”, from which participants needed to choose according to their own experiences. In addition, physiological data of all subjects were collected from 9:00 am to 11:00 am on the test day.

The experimental environment was chosen to be a well-ventilated laboratory with good lighting. During the experiment, the participants were instructed to remain still and silent. After completing one set of experiments, there was a 3 min break, during which the experimenter performed a series of tasks, such as changing the mask and purging the odor channel, to ensure that any residual odor from the previous set did not affect the next set. Additionally, the correctness of the device connection and the accuracy of the physiological data were verified before initiating a new experiment.

### 2.3. Data Processing and Analysiss

#### 2.3.1. Indicator Extraction

**Figure 4:**
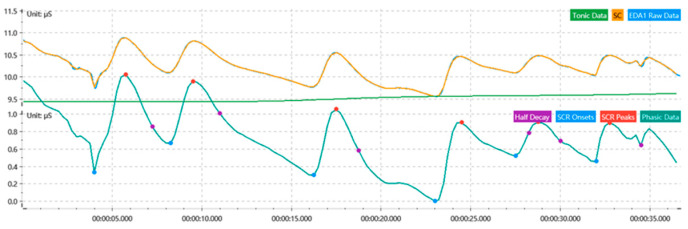
Skin electrical signal processing.

**Figure 5:**
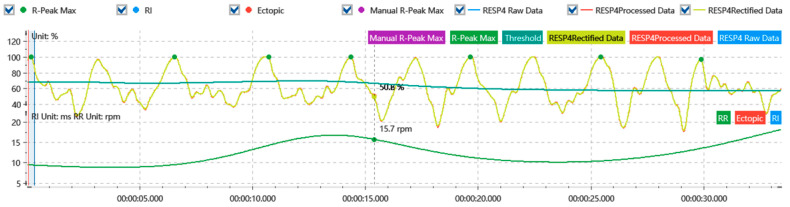
Respiratory signal processing.

**Figure 6:**
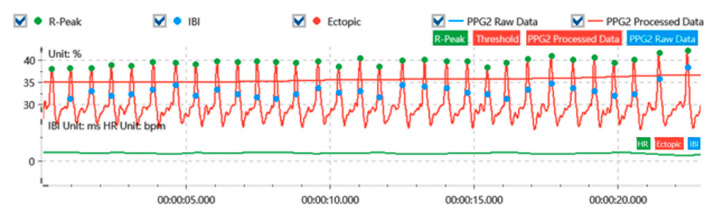
Blood volume pulse signal processing.

The raw signal recordings from physiological sensors are susceptible to noise, artifacts, measurement gaps, and biases. Therefore, preprocessing the raw data to filter noise and remove artifacts is crucial for obtaining stable and reliable physiological signals. For the skin electrical signal, a sliding mean was used to smooth and denoise the signal, and it was filtered using a 5 Hz high-pass filter. Finally, SC was extracted from the time-domain analysis (as shown in Figure 4). For the respiratory signal, the moderate wavelet denoising technique was used, and a low-pass filter with a cutoff frequency of 20 Hz was used for the filtering. Finally, RESP was extracted from the time-domain analysis (as is shown in Figure 5). For the blood volume pulse signal, moderate wavelet denoising was applied, followed by filtering the signal with a 50 Hz band-stop filter, and finally extracting HR from time-domain analysis (as shown in Figure 6).

The signal was segmented according to the four stages designed in the experiment. The mean value of the physiological signal in each stage represents the signal value for that stage. The signal value calculation process for *SC_Mean* is as follows:

(1) $$SC\_Mean=\frac{\sum_{1}^{n}SC}{n}$$

where *SC_Mean* indicates the average value of skin conductivity at a certain stage. *SC* represents the skin conductivity of sampling point at a certain stage. Value of n indicates the number of sampling points. *AVRESP* is calculated as follows:

(2) $$AVRESP=\frac{\sum_{1}^{n}RESP}{n}$$

where *AVRESP* indicates the average value of respiratory rate at a certain stage. RESP represents the respiratory rate of sampling point at a certain stage. Value of n indicates the number of sampling points. *HR_Mean* is calculated as follows:

(3) $$HR\_Mean=\frac{\sum_{1}^{n}HR}{n}$$

where *HR_Mean* indicates the average value of heart rate at a certain stage. *HR* represents the heart rate of sampling point at a certain stage. Value of n indicates the number of sampling points.

#### 2.3.2. Extraction of Physiological Signal Differences

The signal value difference between the calm and stimulating stages is used as the signal change value. The signal value difference for *SC_Mean* is calculated as follows:

(4) $$SC\_Mean’={SC\_Mean}_{ss}-{SC\_Mean}_{cs}$$

where *SC_Mean’* indicates the change value in skin conductance. *SC_Mean_ss_* represents the average of skin conductance during the stimulation stage. *SC_Mean_cs_* indicates the average of skin conductance during the calm stage. The signal value difference for *AVRESP* is calculated as follows:

(5) $$AVRESP’={AVRESP}_{ss}-{AVRESP}_{cs}$$

where *AVRESP’* indicates the change value in respiratory rate. *AVRESPss* represents the average of respiratory rate during the stimulation stage. *AVRESPcs* indicates the average of respiratory rate during the calm stage. The signal value difference for *HR_Mean* is calculated as follows:

(6) $$HR\_Mean’={HR\_Mean}_{ss}-{HR\_Mean}_{cs}$$

where *HR_Mean’* indicates the change in value of heart rate. *HR_Mean_ss_* represents the average of heart rate during the stimulation stage. *HR_Mean_cs_* indicates the average of heart rate during the calm stage.

#### 2.3.3. Preference Comparative Analysis

The focus of the preference comparative analysis was on the physiological signal trends and differences during the “stimulation stage”, when the human body exhibited olfactory likes and disgusts. Preference comparative analysis is divided into overall analysis and individual analysis.

In the overall analysis, the total sample was divided into two categories based on preferences: “like” and “disgust”. The signal change values of individuals in each category were calculated separately. The arithmetic mean of the signal change values for the two categories was computed. The positive and negative signs of the arithmetic mean were interpreted as the average change trend in preferences. The signal change values and average change trends of the two categories were then analyzed to determine the physiological signal change trends when participants exhibited different olfactory perception preferences (like and disgust). A paired sample *t*-test was performed to assess the significance of the change trends, with *p* < 0.05 considered significant.

In the individual analyses, the signal change values for different preferences were extracted for each participant. Participants had to have paired preferences; if a participant only had a signal change value in the “like” state, only the first signal change value in the “disgust” state was extracted. If a participant was exclusively in the same preference state, they were skipped. The signal change values under the two preference states were then compared and analyzed to determine the physiological signal differences when participants exhibit different olfactory perception preferences. A paired sample *t*-test was conducted to evaluate the significance of the signal differences between preferences, with *p* < 0.05 regarded as significant.

#### 2.3.4. Comparative Analysis of Male and Female

There are differences in autonomic responses between different genders when faced with stimuli [38], which may lead to different physiological signals during olfactory perception preferences. Therefore, comparative analysis between genders is crucial. The total sample was divided into two small samples based on gender (male or female). The average signal change values of the two small samples were calculated separately. The average signal change values of the two small samples were analyzed to determine the differences in physiological signal changes between males and females when olfactory perception preferences occurred. Independent sample *t*-tests were performed to determine the significance of differences between different genders, with *p* < 0.05 considered significant.

#### 2.3.5. Prediction of Olfactory Perception Preference

A logistic regression prediction model was established to verify the effectiveness of predicting olfactory perception preferences based on physiological signal changes. In the prediction model, SC_Mean’, AVRESP’, and HR_Mean’ were used as independent variables, and predicted preferences (“like”, “disgust”) were used as dependent variables. The area under the characteristic curve was also statistically analyzed.

## 3. Results

### 3.1. Comparative Analysis Results of Preferences

**Figure 7:**
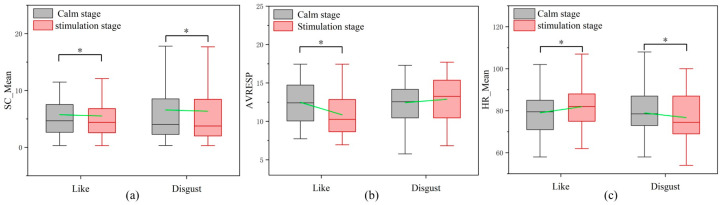
Comparison of SC_Mean, AVRESP, and HR_Mean during the stimulation and calm stage under different preferences. Green line is the average value of the two groups. (**a**) Comparison of SC_Mean during the stimulation and calm stage. (**b**) Comparison of AVRESP during the stimulation and calm stage. (**c**) Comparison of HR_Mean during the stimulation and calm stage. * *p* < 0.05.

A total of 132 samples were divided into two types of small samples (“like”: 66 samples, “disgust”: 66 samples). In the overall analysis, the average trend in skin conductance decreases when the olfactory perception is “like” (*t* = 1.997, *p* < 0.05; 55 samples have an SC_Mean’ of less than zero; the arithmetic mean of the SC_Mean’ for this sample is −0.213; as shown in Table 2 and Figure 7a). The average trend in respiration decreases when the olfactory perception is “like” (*t* = 1.997, *p* < 0.05; 50 samples have an AVRESP’ of less than zero; the arithmetic mean of the AVRESP’ for this sample is −1.629; as shown in Table 2 and Figure 7b). The average trend in heart rate increases when the olfactory perception is “like” (*t* = 1.997, *p* < 0.05; 57 samples have an HR_Mean’ of greater than zero; the arithmetic means of the HR_Mean’ for this sample is 2.893; as shown in Table 2 and Figure 7c).

The average trend in skin conductance decreases when the olfactory perception is “disgust” (*t* = 1.997, *p* < 0.05; 59 samples have an SC_Mean’ of less than zero; the arithmetic mean of the SC_Mean’ for this sample is −0.221; as shown in Table 2 and Figure 7a). The trend in respiration in “disgust” is relatively chaotic (as shown in Table 1 and Figure 7b), with 37 samples having an AVRESP’ of greater than zero (*t* = 2.028, *p* < 0.05; with an arithmetic mean of AVRESP’ of 2.814 for this sample), and 29 samples having an AVRESP’ of less than zero (*t* = 2.048, *p* < 0.05; with an arithmetic mean of AVRESP’ of −2.578 for this sample). The average trend in heart rate decreases when the olfactory perception is “disgust” (*t* = 1.99, *p* < 0.05; 54 samples have an HR_Mean’ of less than zero; the arithmetic means of HR_Mean’ for this sample is −22.181; as shown in Table 2 and Figure 7c).

**Figure 8:**
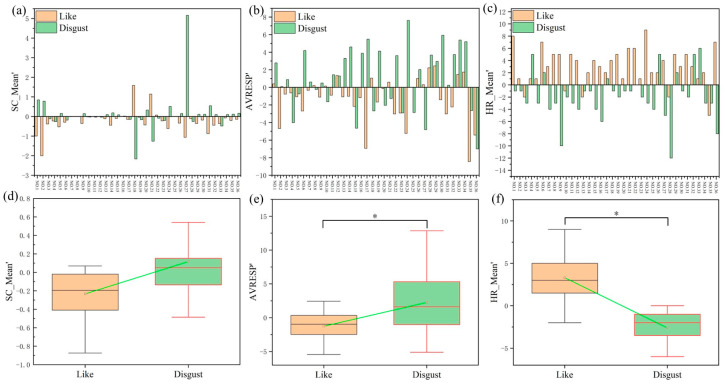
Comparison of individual SC_Mean’, AVRESP’, and HR_Mean’ in “like” and “disgust” states. (**a**–**c**) Variation values of 36 samples in “like” and “disgust” states, where the abscissa represents the number of the 36 samples selected. (**d**–**f**) Statistical chart of 36 samples in “like” and “disgust” states. (**a**) corresponds to (**d**) and so on. Green line is the average value of the two groups. * *p* < 0.05.

In the individual analyses, 36 samples were selected from 132 samples. A total of 20 samples had a lower SC_Mean’ in the ‘‘like’‘ state than that in the “disgust” state (as shown in Figure 8a), but there was no statistically significant difference (as shown in Figure 8d). Additionally, 25 samples showed a lower AVRESP’ in the ‘‘like’‘ state than in the ‘‘disgust’‘ state (*t* = 2.030, *p* < 0.05; as shown in Figure 8b,e). In 23 samples, the HR_Mean’ was greater than zero in the ‘‘like’‘ state and less than zero in the ‘‘disgust’‘ state (*t* = 1.994, *p* < 0.05; as shown in Figure 8c,f).

### 3.2. Comparative Analysis Results of Male and Female

**Figure 9:**
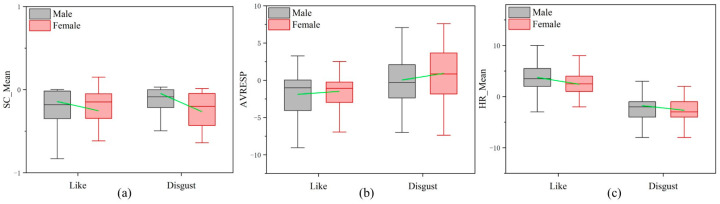
Comparison of SC_Mean’, AVRESP’, and HR_Mean’ between males and females in the “like” and “disgust” states. (**a**) Comparison of SC_Mean’ between males and females. (**b**) Comparison of AVRESP’ between males and females. (**c**) Comparison of HR_Mean’ between males and females. Green line is the average value of the two groups. * *p* < 0.05.

In the “like” state, the SC change trend in the males is less than that in the females (the arithmetic mean of the SC_Mean’ in the male samples is −0.142, and the arithmetic mean of the SC_Mean’ in the female samples is −0.253; as shown in Table 2 and Figure 9a), while the RESP change trend is greater than that in the females (the arithmetic mean of the AVRESP’ in the male samples is −1.877, and the arithmetic mean of the AVRESP’ in the female samples is −1.487; as shown in Table 3 and Figure 9b). The HR change trend is greater than that in the female samples (the arithmetic mean of the HR_Mean’ in the male samples is 3.750, and the arithmetic mean of the HR_Mean’ in the female samples is 2.404; as shown in Table 3 and Figure 9c). However, there is no statistically significant difference between the above signal change values.

In the “disgust” state, the SC change trend in the males is less than that in the females (the arithmetic mean of the SC_Mean’ in the male samples is −0.046, and the arithmetic mean of the SC_Mean’ in the female samples is −0.264; as shown in Table 3 and Figure 9a), while the RESP change trend is less than that in the female samples (the arithmetic mean of the AVRESP’ in the male samples is 0.036, and the arithmetic mean of the AVRESP’ in the female samples is 0.935; as shown in Table 3 and Figure 9b). The HR change trend is less than that in the female samples (the arithmetic mean of the HR_Mean’ in the male samples is −1.777, and the arithmetic mean of the HR_Mean’ in the female samples is −2.666; as shown in Table 3 and Figure 9c). However, there is no statistically significant difference between the above signal change values.

### 3.3. Prediction Results of Olfactory Perception Preference

**Figure 10:**
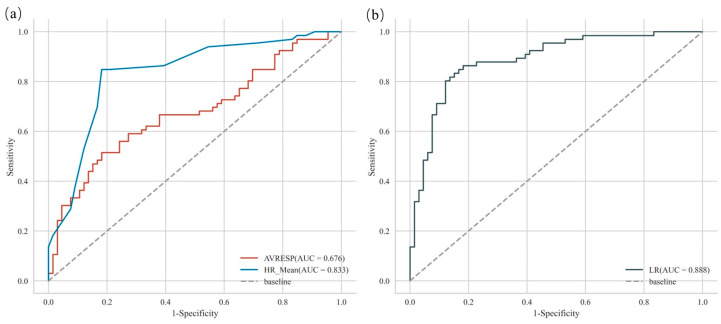
The Receiver Operating Characteristic Curve (ROC) of the logistic regression model. (**a**) The Receiver Operating Characteristic Curve (ROC) under univariate input. (**b**) The Receiver Operating Characteristic Curve (ROC) under multivariate input. AUC refers to the area enclosed by the curve and the X and Y axes.

This study used the SK-Learn toolkit (v1.2.2) and the PyCharm (v2022.2.3) interpreter to build a logistic regression model. We used 144 samples that were collected in the experiment to test our model, with 70% of the samples used for training the model and 30% of the samples used for testing the model. The model results show that the increase in the SC_Mean’ is correlated with the prediction of a preference for “like”, but the effect does not reach statistical significance (as shown in Table 4). The decrease in the AVRESP’ is correlated with the prediction of preference for “like” (*p* < 0.05; as shown in Table 4), and the increase in the HR_Mean’ is correlated with the prediction of preference for “like” (*p* < 0.05; as shown in Table 4). The areas under the curves (AUCs) of the models are 0.676 and 0.833, when the AVRESP’ and HR_Mean’ are used as the input features of the model, respectively (as shown in Figure 10a). Therefore, the influence of the HR_Mean’ on the model is greater than that of the AVRESP’. When the two are combined as the inputs, the model’s AUC was 0.888 (as shown in Figure 10b). Therefore, when the AVRESP’ and HR_Mean’ are combined as the inputs for the model, the model’s prediction accuracy is optimal (the model’s prediction accuracy was 84.1%; as shown in Table 5).

## 4. Discussion

Skin conductance, respiration, and heart rate are commonly used methods for assessing physiological status. They are usually considered as indicators related to the activity of the autonomic nervous system, and are widely used in the fields of psychophysiology, emotion research, and physiological state monitoring. This study aims to investigate the autonomic response (skin conductance, respiration, and heart rate) of fragrance consumers when they exhibit olfactory preferences. The main results of this paper are as follows.

Olfactory preferences can lead to changes in SC, RESP, and HR. These findings are consistent with previous literature, confirming that changes in physiological signals can be used to assess olfactory preferences [39]. The change trends in the RESP and HR are statistically different when the olfactory preferences differ (*p* < 0.05). However, the change trend in the SC is the same when the olfactory preferences differ. This conclusion is consistent with that obtained from the overall analysis and individual analyses. The change trend in the SC and RESP are decreasing, while the change trend in the HR is increasing (*p* < 0.05) when the olfactory perception is “like”. The change trend in the SC is the same as that in the “like” state when the olfactory perception is “disgust”. Although there are numerical differences in the change trend in the SC under different preferences, nevertheless there is no statistical difference. This indicates that the SC change values of consumers during sniffing cannot accurately distinguish between olfactory likes and disgusts. The trend in the RESP changes in the state of “disgust” is more chaotic (*p* < 0.05). But in individual analysis, 25 samples showed lower RESP changes in the state of “like” than in the state of “disgust” (*p* < 0.05). This indicates that there are differences in the RESP changes among different preferences. Therefore, the RESP changes can provide a reference for distinguishing between olfactory likes and disgust. The trend in change in the HR varies under different preferences. Moreover, in individual analyses, 23 samples exhibit significant differences (*p* < 0.05). Therefore, HR changes can effectively distinguish olfactory preferences. Compared to SC and RESP, HR is more suitable for evaluating olfactory preferences.

Our findings appear to contradict previous reports, which noted that the SC increases during the preference period and that the changes are significantly different [26]. However, this scheme involved selecting preferred products using buttons, with no response to non-preferred products. Nevertheless, in our study, participants did not engage in any operations that would interfere with the physiological signals of skin conductance. The action of pressing the button generates a larger SC, which may be a factor contributing to the inconsistency with the results presented in this paper. Another study reported results similar to those of this paper, but it noted that the changes in SC during the preference period showed significant differences [40]. However, this report used visual stimuli to investigate the SC during the preference period. Generally, visual stimuli tend to elicit stronger skin conductance responses because visual information is processed more quickly and has a more direct connection to emotional responses [41]. This could be a possible reason for the significant differences observed in the SC changes.

Another possibility is that olfactory preferences lead to changes in consumers’ emotional states. People tend to become excited and joyful in a “like” state, while their emotions deteriorate and become low in a “disgust” state. Researchers have demonstrated that positive emotions lead to an increase in HR, while negative emotions lead to a decrease [42]. Additionally, there are significant differences in HR between positive and negative emotions, while there is no significant correlation between SC and emotions [43,44]. RESP typically decreases with pleasant emotions. However, negative emotions often lead to irregular breathing patterns [45].

In the comparison of the physiological signal changes between the males and females, the trends in the SC, RESP, and HR changes are consistent with the overall analysis. The specific differences between the genders are as follows: (1) Regardless of whether in a “like” or “disgust” state, the average change trend in the SC in the females is greater than in the males. (2) In the “like” state, the average change trend in the RESP in the males is greater than in the females, while in the “disgust” state, it is less than in the females. (3) In the “like” state, the average change trend in the HR in the males is greater than in the females, while in the “disgust” state, it is less than in the females. However, none of these differences demonstrate statistical significance. Therefore, there are no significant differences in the physiological signal changes between the males and females when olfactory preferences are present, and the autonomic responses in the males and females are consistent. This is contrary to previous research findings [46], which showed that due to differences in sex hormone levels, males and females exhibit different autonomic nervous responses to stimuli. The reason for the difference in this current study from these previous findings may be that the olfactory stimuli in this study are relatively mild and do not reach the stimulus levels in the previous research, resulting in no significant differences in the physiological signal changes between the males and females in this study.

The olfactory preference prediction model we established has a high accuracy rate. However, the influence of the SC_Mean’ on the model does not reach statistical significance. Therefore, the effect of using SC to predict olfactory perception preferences is very poor, which is consistent with the conclusions drawn from the statistical analyses. In addition, the AUC results show that the HR_Mean’ has a greater impact on the model than the AVRESP’. When the AVRESP’ and the HR_Mean’ are combined as the model inputs, the model’s AUC is the highest. Therefore, this is the best scheme for predicting olfactory perception preferences when using AVRESP’ and HR_Mean’ joint evaluation metrics.

However, this study still has limitations. Firstly, preferences were only categorized into two groups, without distinguishing between different levels of preference. Therefore, the differences in the physiological signals within the same preference category remain unknown. Secondly, this study only collected autonomic responses from a single age group, and future work can explore the autonomic responses of consumers from different age groups and even different ethnicities. Thirdly, there is a wide variety of indicators for physiological signals; however, only SC, RESP, and HR were utilized in our studies. Future research can investigate the correlation between other physiological signals (e.g., electro-oculogram, skin temperature, and galvanic skin response) as well as olfactory perception preferences. Finally, the regression model we used is simple, efficient, and suitable for exploratory research. However, its prediction accuracy may not be as good as other advanced models. Logistic regression was used to establish a predictive model in this study, and other algorithms can be employed to improve accuracy in predicting preferences in future studies. Despite the aforementioned limitations, it is important to note that this current study serves as a preliminary investigation into this field. This study reveals the correlation between commonly used physiological signals and consumer olfactory preferences, providing a solid foundation for the assessment of olfactory preferences through physiological indicators.

## 5. Conclusions

In summary, we have preliminarily identified autonomic responses (skin conductance, respiration, and heart rate) in consumers with olfactory perception preferences. The results of this study have significant practical implications for the field of fragrance products, particularly in fragrance product development and marketing. Utilizing physiological signals can help manufacturers better understand consumers’ needs and preferences during the early design phase of fragrances, ultimately saving time and costs. Additionally, the olfactory preferences of consumers are more accurately expressed through physiological signals. Therefore, customized fragrance designs are more easily established, and personalized olfactory experiences can be offered to different types of consumers through customized fragrance designs, enhancing the product’s competitiveness and appeal.

## Figures and Tables

**Table 1:** Participant Screening Form. Functional issues were selected, and other privacy issues were not given. Individuals that satisfied all screening questions were selected as participants.

Test Project	Agree	Disagree
I don’t have any neurological diseases.		
I don’t have rhinitis.		
I can accurately perceive smells.		
I am able to clearly express what I want.		
I often use fragrance products in my daily life (more than twice a week).		
I’m not allergic to any smell.		

**Table 2:** The comparison of the SC_Mean, RESP, and HR_Mean between the calm and stimulated stages for the “like” and “disgust” states. The values in the calm and stimulated phases are arithmetic averages.

Change Value	Calm Stage (Like)	Stimulation Stage (Like)	*t*	*p*
*SC_Mean*	5.736	5.523	1.997	<0.01
*AVRESP*	12.480	10.851	1.997	<0.01
*HR_Mean*	79.030	81.924	1.997	<0.01
**Change Value**	**Calm Stage (Disgust)**	**Stimulation Stage (Disgust)**	***t***	***p***
*SC_Mean*	6.570	6.348	1.997	<0.01
*AVRESP*	12.436	12.881	1.997	0.286
*HR_Mean*	78.924	76.742	1.997	<0.01

**Table 3:** Comparison of SC_Mean’, AVRESP’, and HR_Mean’ between males and females in the “like” and “disgust” states. Like and disgust are both arithmetic means.

Change Value	Like (Male)	Like (Female)	Difference (Absolute Value)	*t*	*p*
*SC_Mean’*	−0.142	−0.253	0.111	2.028	0.382
*AVRESP’*	−1.877	−1.487	0.39	1.997	0.614
*HR_Mean’*	3.75	2.404	1.346	2.030	0.209
**Change Value**	**Disgust (Male)**	**Disgust (Female)**	**Difference (Absolute Value)**	***t***	***p***
*SC_Mean’*	−0.046	−0.264	0.218	2.015	0.141
*AVRESP’*	0.036	0.935	0.899	1.997	0.283
*HR_Mean’*	−1.777	−2.666	0.889	1.997	0.350

**Table 4:** The results of the logistic regression model. The influence of Intercept and SC_Mean’ on the model did not reach statistical significance, so their AUC were not provided. β refers to the coefficient in the regression equation provided in the footnote.

Prediction Model	Independent Variable	β	*p*	AUC
Logistic regression	*Intercept*	−0.329	0.190	\
	*SC_Mean’*	0.039	0.921	\
	*AVRESP’*	−0.335	<0.01	0.676
	*HR_Mean’*	0.474	<0.01	0.833

$g_{preference}=\frac{1}{1+e^{\beta_{intercept}+\beta_{SC\_Mean}*SC\_Mean+\beta_{RESP}*RESP+\beta_{HR\_Mean}*HR\_Mean}}$.

**Table 5:** Confusion matrix of the model under multivariable inputs (*AVRESP’* and *HR_Mean’*).

Observation/Forecast	Forecast		
Observation	Like	Disgust	Correct Percentage
Like	55	11	83.3%
Disgust	10	56	84.8%
Overall percentage			84.1%

## Data Availability

The data segments can be obtained by contacting the corresponding author.
